# Thermal care of newborns: drying and bathing practices in Malawi and Bangladesh

**DOI:** 10.7189/jogh.08.010901

**Published:** 2018-06

**Authors:** Shane M Khan, Eunsoo Timothy Kim, Kavita Singh, Agbessi Amouzou, Liliana Carvajal-Aguirre

**Affiliations:** 1 Data and Analytics, Division of Data, Research and Policy, United Nations Children’s Fund (UNICEF), New York, New York, USA; 2 Department of Maternal and Child Health, Gillings School of Global Public Health, University of North Carolina at Chapel Hill, Chapel Hill, North Carolina, USA; 3 Carolina Population Center, University of North Carolina at Chapel Hill, Chapel Hill, North Carolina, USA; 4 Johns Hopkins Bloomberg School of Public Health, Baltimore, Maryland, USA

## Abstract

**Background:**

Thermal care of newborns is one of the recommended strategies to reduce hypothermia, which contributes to neonatal morbidity and mortality. However, data on these two topics have not been collected at the national level in many surveys. In this study, we examine two elements of thermal care: drying and delayed bathing of newborns after birth with the objectives of examining how two countries collected such data and then looking at various associations of these outcomes with key characteristics. Further, we examine the data for potential data quality issues as this is one of the first times that such data are available at the national level.

**Methods:**

We use data from two nationally-representative household surveys: the Malawi Multiple Indicator Cluster Survey 2014 and the Bangladesh Demographic and Health Survey 2014. We conduct descriptive analysis of the prevalence of these two newborn practices by various socio-demographic, economic and health indicators.

**Results:**

Our results indicate high levels of immediate drying/drying within 1 hour in Malawi (87%). In Bangladesh, 84% were dried within the first 10 minutes of birth. Bathing practices varied in the two settings; in Malawi, only 26% were bathed after 24 hours but in Bangladesh, 87% were bathed after the same period. While in Bangladesh there were few newborns who were never bathed (less than 5%), in Malawi, over 10% were never bathed. Newborns delivered by a skilled provider tended to have better thermal care than those delivered by unskilled providers.

**Conclusion:**

These findings reveal gaps in coverage of thermal care and indicate the need to further develop the role of unskilled providers who can give unspecialized care as a means to improve thermal care for newborns. Further work to harmonize data collection methods on these topics is needed to ensure comparable data across countries.

Globally, under-five mortality has declined between 1990 and 2015, from 91 deaths per 1000 live births to 43 deaths per 1000 live births [1]. While neonatal mortality also declined during this period, the share of neonatal deaths among under-five deaths increased to approximately 45% in 2015 [1]. In the neonatal period (the first 28 days of life), nearly 1 million newborns die on the first day (37% of all neonatal deaths) and approximately 2 million newborns die in the first week (74% of all neonatal deaths) [1,2]. Of these deaths, the majority are preventable using simple and effective life-saving interventions [3].

The “Every Newborn Action Plan” which is supported by the World Health Organization (WHO) and the United Nations Children’s Fund (UNICEF) and various other institutions emphasizes the critical period of labour, birth and the first week of life as a period that can be targeted to prevent newborn deaths [4]. An important set of interventions designed to minimize preventable deaths during this critical period is essential newborn care (ENC), which broadly includes hygienic cord care, thermal protection and early initiation of breastfeeding [4]. Thermal protection and care of newborns is recommended as it reduces hypothermia, a condition in which the body temperature falls below normal levels [5] and which is known to contribute to global neonatal mortality either directly or indirectly as a comorbidity of other major causes of death [6]. A recent systematic review of low- and middle-income countries found that the prevalence of hypothermia ranged between 32% and 85% for newborns delivered at hospitals and between 11% and 92% for newborns delivered at homes [6]. The review suggests that the wide-ranging prevalence of hypothermia may be associated with different risk factors including low environmental temperatures, early bathing, low socioeconomic status of the mother/family and newborn complications such as low birth weight, prematurity, intrauterine growth restriction and birth asphyxia [6].

Key interventions to prevent newborn hypothermia and its associated mortality risk are described as a chain of interlinked operations. The WHO practical guide for thermal protection of newborns recommends several key interventions to ensure that the newborn is kept warm. This includes that the place of delivery is warm, newborns are immediately dried and either wrapped or placed on the mother for skin-to-skin contact. The recommendation also states that bathing should be delayed for at least 24 hours following birth, or six hours, if culturally appropriate, and that breastfeeding is initiated within one hour after delivery [5].

A 2013 WHO recommendation on postnatal thermal care for newborns refers to the timing of first bath and echoes the same recommendation the timing of bathing [7]. The integrated management of pregnancy and childbirth provides similar advice on drying and placing the baby on the mother’s chest with skin-to-skin contact right after birth [8]. In addition, the recently released “Standards for improving quality of maternal and newborn care in health facilities” [9] includes immediate and thorough drying of the newborn as a quality measure. Drying and rubbing are also an essential step in newborn resuscitation used with newborns who do not breathe spontaneously after birth [9].

Several studies in the past have examined the use of protective thermal care practices for newborns. Many have found that there is low use of adequate thermal care practices across South Asia and sub-Saharan Africa. For example, Pagel et al. reports that newborn wrapping or skin-to-skin contact within 10 minutes was relatively low for both home and facility deliveries in Eastern India and Bangladesh [10]. Coverage of bathing after 6 hours, varied widely by study location and delivery types [10]. Another study in Bangladesh reports that only 5.1% of newborns received complete thermal protection, defined as drying and wrapping within 10 minutes and bathing after 72 hours of birth [11].A study in western Uganda had higher proportions of wrapping the newborn (85.1%) and delaying bathing until after 24 hours (66.3%) [12]. Key findings from qualitative and mixed-method studies have also shown that newborn bathing practices varied across studies but were mostly favouring early bathing due to strong cultural and traditional beliefs [13-16].

Among the recommended thermal care practices, there are few countries with national data on the topics. However, two recent national surveys in Malawi and Bangladesh have included new questions on newborn drying and bathing. There are two primary objectives of this study. The first is to describe the approaches used to collect data on these topics and then to describe associations between these two thermal care practices and key variables. As this is one of the first time that these data are being examined at the national level in the Malawi Multiple Indicator Cluster Survey (MICS) 2014 and the Bangladesh Demographic and Health Survey (DHS) 2014, we also examine potential data quality issues which can inform the development of future data collection on drying and bathing using household surveys.

## METHODS

### Data source

We searched the UNICEF-supported Multiple Indicator Cluster Surveys (MICS) and the USAID-supported Demographic and Health Surveys (DHS) for data on newborn drying and bathing. Data were available from the Malawi MICS 2014 and the Bangladesh DHS 2014. The surveys used a similar two-stage sampling methodology, where census enumeration areas were first selected and then households were selected in the second stage. In the households, all women age 15-49 were interviewed in Malawi while in Bangladesh, only ever-married women were interviewed. In the Malawi MICS 2014, questions on drying and bathing were asked about the last birth in the last 2 years, while in Bangladesh, questions were asked about the last birth in the last 3 years. The sample sizes were 7490 in Malawi and 4626 in Bangladesh.

### Questions on drying

The Malawi MICS 2014 asked “Was (name) dried or wiped after delivery?” The question provided response categories of ‘yes’, ‘no’, ‘don’t know’ and ‘missing’ and among those who said yes, “How soon after birth was (name) dried or wiped?” recording the time in hours, with additional categories for immediate drying/less than 1 hour and another category for don’t know/don’t remember.

The Bangladesh DHS 2014 asked one question on drying, “How long after birth was (NAME) dried?” providing categorical response categories of <5 minutes, 5-9 minutes, 10+ minutes, not dried, and don’t know.

### Questions on bathing

The question on bathing in the Malawi MICS 2014 was, “How soon after birth was (name) bathed for the first time?” recording the time in hours, with additional categories for immediate bathing (less than an hour), never bathed and don’t know/don’t remember. It should be noted that in this survey, newborns who were not dried were skipped out of the question on bathing.

The Bangladesh DHS 2014 asked, “How long after delivery was (NAME) bathed for the first time?” recording the time in hours (if less than 1 day), days (if less than 1 week), weeks, not bathed, and don’t know.

### Variables and analysis

As the aim of this analysis is to understand patterns of drying and bathing across the two countries, the analysis is descriptive. Questions on drying are not comparable across the two surveys and do not allow the calculation of a comparable ‘immediate drying’ variable. Therefore, in both data sets, we dichotomized the variable as ‘dried’ or ‘not dried’ without regard for the timing of drying. Missing and don’t know cases were minimal and treated as “not bathed” in **Table 1** and **Table 2**.

**Table 1:** Percentage of newborns which were dried in Malawi, by place of delivery and various characteristics

	Place of delivery					
	Facility	N	Non-facility	**N**	**Total**	**N**
**Mother's age (years):**						
<20	91.3	947	93.4	55	91.4	1002
20-34	93.5	4962	81.8	413	92.6	5375
35-49	91.3	968	86.5	145	90.7	1113
**Birth order:**						
1	94.2	152	94.9	16	94.3	168
2-3	91.2	553	82.7	57	90.4	610
4-5	92.9	1489	87.5	116	92.5	1605
6+	93.1	4683	82.7	424	92.2	5107
**Mother's education:**						
No education	92.3	743	77.2	129	90.1	872
Primary education	93.0	4866	85.5	453	92.3	5318
Secondary or higher	92.9	1268	89.3	32	92.8	1300
**Size at birth:**	‡					
Very small	92.7	431	61.8	61	75.2	492
Smaller than average	93.9	584	84.9	66	93.0	650
Average	94.6	3495	85.0	325	93.8	3820
Larger than average	93.2	1684	84.5	111	92.7	1795
Very large	93.6	683	87.5	50	93.2	733
**Attendant at delivery:**	‡				‡	
Skilled	94.5	6383	100.0	610	94.5	6386
Unskilled provider	71.6	494	90.1	3	78.4	1104
**C-section:**	‡				‡	
No	93.6	6482	84.0	515	92.8	7095
Yes	81.3	396	0.0	0	81.3	396
**Residence:**	†		‡		‡	
Urban	88.8	860	45.6	29	87.4	889
Rural	93.5	6018	85.9	584	92.8	6602
**Wealth:**			‡			
Poorest	93.2	1615	88.3	237	92.5	1853
Second	94.5	1522	87.5	153	93.8	1676
Middle	92.7	1425	82.0	132	91.8	1556
Fourth	92.3	1176	72.5	67	91.2	1242
Richest	91.2	1139	61.7	24	90.6	1163
**Antenatal visits:**	‡				‡	
None	43.9	169	75.6	68	53.3	237
1	95.8	133	83.7	41	92.9	174
2	94.5	755	84.7	121	93.1	877
3	93.1	2650	84.1	202	92.4	2852
4+	94.8	3170	86.2	181	94.4	3351
**Total**	92.9	6877	84.0	613	92.2	7490

*P < 0.05.

†*P* < 0.01.

‡*P* < 0.001.

**Table 2:** Percentage of newborns which were dried in Bangladesh, by place of delivery and various characteristics

	Place of delivery					
	**Facility**	**N**	**Non-facility**	**N**	**Total**	**N**
**Mother's age (years):**						
<20	89.7	351	93.1	619	91.8	970
20-34	89.9	1334	92.1	2043	91.2	3379
35-49	82.4	100	84.1	178	83.5	277
**Birth order:**			†		*	
1	89.0	888	93.8	956	91.4	1845
2-3	90.4	786	91.3	1355	91.0	2141
4-5	87.7	96	93.2	388	92.1	484
6+	77.5	14	79.0	141	78.8	155
**Mother's education:**			†		*	
No education	85.7	283	89.1	1120	88.4	1403
Primary education	89.8	955	93.1	1479	91.8	2436
Secondary or higher	90.8	545	96.1	241	92.4	786
**Size at birth:**						
Very small	86.3	124	91.4	191	89.4	316
Smaller than average	85.6	198	88.0	409	87.2	607
Average	90.8	1193	92.4	1911	91.8	3105
Larger than average	88.4	240	91.6	256	90.1	495
Very large	82.2	29	97.4	73	93.1	102
**Attendant at delivery:**						
Skilled	89.3	1740	92.9	228	89.7	1970
Unskilled provider	95.3	44	91.7	2612	91.8	2656
**C-section:**					†	
No	92.8	663	91.8	2840	92.0	3504
Yes	87.5	1120	0.0	0	87.4	1121
**Residence:**						
Urban	91.3	705	93.1	504	92.0	1209
Rural	88.3	1078	91.5	2337	90.5	3416
**Wealth:**				†	*	
Poorest	79.8	154	89.3	849	87.8	1003
Second	87.8	214	88.1	660	88.0	876
Middle	88.0	307	94.2	574	92.0	881
Fourth	90.8	455	95.8	500	93.4	955
Richest	92.0	654	96.6	258	93.3	912
**Antenatal visits:**			*		*	
None	87.6	110	88.3	886	88.2	996
1	91.1	219	90.2	608	90.4	827
2	92.5	313	93.8	435	93.2	748
3	90.2	297	96.6	315	93.5	613
4+	87.9	845	94.7	595	90.7	1442
**Total**	89.4	1784	91.8	2840	90.9	4626

*P < 0.05.

†*P* < 0.01.

For the bathing variables, we created a variable to measure bathing after 24 hours. These data were only collected in hours in the Malawi MICS 2014, and were categorized according to the definition of above 24 hours, less than 24 hours, and cases of never bathed, don’t know or missing were coded as missing for the tables. For the Bangladesh DHS 2014, we coded bathing at day 1 as bathing after 24 hours (as values over 24 hours were collected in days), coding not bathed, don’t know and missing as missing values for the tables. We stratified the analysis by facility vs non-facility births given that the recommendations on thermal care can theoretically differ across these strata. In both surveys, facility births included hospitals, clinics and health centres while non-facility births were all other locations.

In the analysis, we used a number of background variables to examine drying and bathing. Mother’s age in years was categorized as less than 20, 20 to 34 and 35 to 49. Birth order referred to the order of the birth of the newborn, categorized as 1 which refers to the first birth, 2-3 (the second and third births), 4-5 (fourth and fifth births) and 6+ which referred to sixth and higher order births. Mother’s education was categorized as no education, primary, and a third category of secondary and higher (including university). Size of birth is based on the mother’s perception of how large or small the newborn was (at birth). Attendant at delivery was categorized as either skilled or unskilled. Skilled in Malawi referred to births delivered by a doctor or nurse/midwife while skilled in Bangladesh referred to births delivered by a qualified doctor, nurse/midwife/paramedic while unskilled referred to the residual categories in each country. C-section referred to Caesarean section for delivery of the newborn. Residence referred to the type of location where the woman lived and was categorized as urban or rural. The wealth variable was calculated using Principal Component Analysis (PCA). The PCA assigned overall scores to households based on household ownership of goods and assets. The PCA ranked households and then categorized them into five categories: poorest, second, middle, fourth and richest households. Antenatal care referred to the number of times a woman visited any provider during the pregnancy of the newborn for antenatal care. This variable was categorized into no visits (labelled “none”), 2, 3 and 4 or more visits (labelled “4+”).

Sampling weights supplied with the data sets were used in all analyses.

## RESULTS

**Figure 1:**
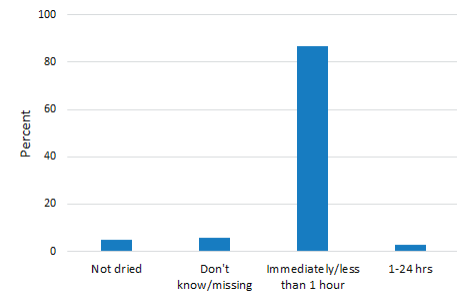
Newborn drying in Malawi.

**Figure 2:**
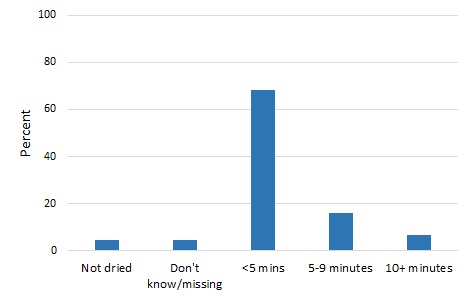
Newborn drying in Bangladesh.

**Figure 1** and **Figure 2** present the distribution of newborn drying in the two countries. An overwhelming majority of women (87%) said that their newborns were dried immediately or within 1 hour in Malawi. In Bangladesh, 68% of women said that their newborns were dried within 5 minutes of birth, 16% within 5-9 minutes following birth and 7%, 10 minutes or later. The percentage of newborns not dried was 5% and 6% in Malawi and Bangladesh respectively.

**Table 1** and **Table 2** show distributions of drying of the newborn in each country and how they vary by facility and key variables. About 90% of newborns were dried in both countries. With such high coverage, differentials across socio-demographic subgroups were generally weak. However, several variables showed notable differences. While newborns who were born in a facility in Malawi were significantly more likely to be dried compared to those born outside a facility (93% vs 84%), in Bangladesh, there is no discernible difference. Likewise, in Malawi, births with a skilled provider were more likely to be dried (95%) compared to those with an unskilled provider (78%) though this pattern was not found in Bangladesh. Women who had had no antenatal visits were least likely to have a newborn that was dried (53%) compared to others with such care (1 or more visits, >90%) in Malawi. This differential was significant in Bangladesh as well but differences in levels were not substantial. In both countries, women who had a C-section reported less drying than women with vaginal births (eg, in Bangladesh, 87% vs 92%). In addition, in Malawi, place of residence was also a marker for differences in drying with a larger proportion of mothers in rural areas reporting that the baby was dried immediately after birth. In Bangladesh, mother’s education and household wealth also show significant differences in the results regarding newborn drying with mothers with no education and in the poorest households reporting lower levels of newborn drying. Despite significance, differences are not substantial.

**Figure 3:**
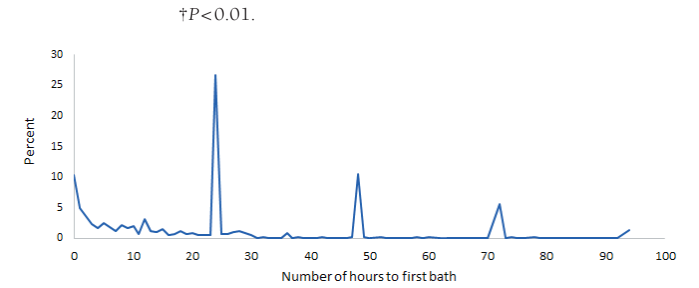
Bathing of newborns in Malawi in hours.

**Figure 4:**
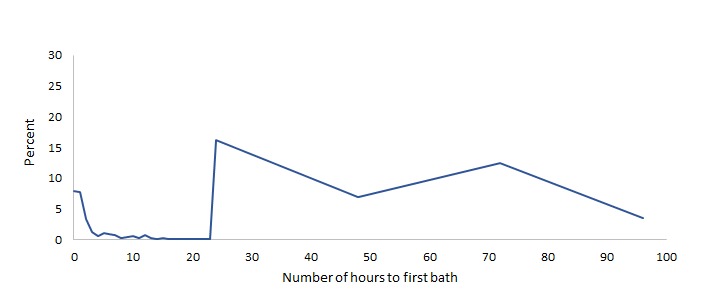
Bathing of newborns in Bangladesh in hours.

We examined the distribution of timing of bathing of newborns for the first 100 hours of life, shown in **Figure 3** and **Figure 4** (excluding cases where newborns were not bathed, about 10% in Malawi and 5% in Bangladesh, or were missing). In both countries, there is a small but important percentage of newborns who are bathed immediately after birth (about 10% in Malawi and 8% in Bangladesh). The graphs also show clear evidence of heaping of the data. In Malawi, this is evident on hours 24, 48 and 72 which correspond to 1, 2 and 3 days. Similarly, in Bangladesh, some heaping occurs at 24 and 72 hours.

**Table 3** and **Table 4** show bathing after 24 hours in Malawi and Bangladesh. In these tables, cases of never bathed, don’t know and missing were removed from analysis. In Malawi, only 26% of newborns were bathed after 24 hours, while in Bangladesh, 74% were bathed after 24 hours. In both countries, there were few missing cases (not included in the **Tables 3** and **4**). Comparing by place of birth, newborns in facilities were more likely to have a delayed bathed compared to those born outside of facilities in both countries (see **Table 3** and **4**). Newborns in Malawi and Bangladesh who were born using a skilled provider or those who were born with a C-section were more likely to have a delayed bath than those who were born to an unskilled provider or those born by a vaginal birth. For example, in Bangladesh, among all newborns, 92% born using a skilled attendant was bathed after 24 hours while only 60% born without a skilled attendant was bathed after 24 hours.

**Table 3:** Bathing of newborns after 24 h in Malawi by place of delivery

	Place of delivery					
	**Facility**	**N**	**Non-facility**	**N**	**Total**	**N**
**Mother's age (years):**						
<20	28.4	738	18.3	45	27.8	783
20-34	27.5	3925	13.5	299	26.5	4224
35-49	25.0	722	10.7	119	23.0	840
**Birth order:**						
1	21.8	118	0.0	13	19.7	131
2-3	22.2	438	7.4	39	21.0	477
4-5	29.4	1190	12.8	89	28.3	1279
6+	27.4	3638	14.6	322	26.4	3960
**Mother's education:**						
No education	26.7	550	20.3	83	25.9	634
Primary education	27.8	3818	11.4	353	26.4	4171
Secondary or higher	25.7	1016	15.8	26	25.4	1042
**Size at birth:**					*	
Very small	34.8	253	6.2	43	30.7	296
Smaller than average	35.4	441	18.1	53	33.6	494
Average	25.2	2756	12.3	241	24.1	2997
Larger than average	28.6	1393	16.8	85	27.9	1477
Very large	24.8	542	12.2	42	23.9	583
**Attendant at delivery:**					‡	
Skilled	27.6	5075	0.0	3	27.6	5078
Unskilled provider	22.7	309	13.3	459	17.1	769
**C-section:**	‡				‡	
No	26.7	5131	0.0	0	25.6	5594
Yes	40.4	254	13.2	462	40.4	254
**Residence:**						
Urban	24.1	662	0.0	11	23.7	674
Rural	27.8	4722	13.6	451	26.5	5174
**Wealth:**			†			
Poorest	24.7	1252	13.5	190	23.3	1442
Second	30.5	1197	7.0	119	28.4	1316
Middle	28.8	1116	21.8	95	28.2	1211
Fourth	25.9	938	5.5	44	25.0	982
Richest	26.2	883	29.3	14	26.3	897
**Antenatal visits:**			*			
None	26.9	43	5.8	47	15.8	90
1	21.6	99	24.8	31	22.4	130
2	29.4	597	12.0	89	27.1	686
3	28.2	2094	17.2	150	27.4	2244
4+	26.3	2552	9.9	146	25.4	2697
**Total**	27.3	5385	13.2	462	26.2	5847

*P < 0.05.

†*P* < 0.01.

‡*P* < 0.001.

**Table 4:** Bathing of newborns after 24 h in Bangladesh by place of delivery

	Place of delivery					
	**Facility**	**N**	**Non-facility**	**N**	**Total**	**N**
**Mother's age (years):**			‡		†	
<20	94.5	337	61.0	605	73.0	942
20-34	94.9	1285	62.1	2009	74.9	3295
35-49	96.4	97	41.7	176	61.1	273
**Birth order:**			‡			
1	95.2	854	62.6	934	78.2	1789
2-3	94.3	760	62.6	1334	74.1	2094
4-5	95.7	92	58.7	381	66.0	473
6+	100.0	13	33.3	141	39.0	154
**Mother's education:**			‡		‡	
No education	95.1	272	51.2	1101	59.9	1373
Primary education	94.1	918	66.0	1452	76.9	2370
Secondary or higher	96.1	530	70.8	237	88.3	767
**Size at birth:**					†	
Very small	91.3	117	59.5	188	71.8	305
Smaller than average	93.8	190	57.1	402	68.8	592
Average	95.5	1164	61.2	1882	74.3	3046
Larger than average	95.1	224	67.2	247	80.5	471
Very large	88.6	25	43.9	71	55.6	96
**Attendant at delivery:**					‡	
Skilled	94.9	1676	67.6	226	91.7	1903
Unskilled provider	92.9	44	59.9	2564	60.5	2607
**C-section:**	‡				‡	
No	90.0	644	-	-	66.1	3434
Yes	97.8	1075	-	-	97.8	1076
**Residence:**					‡	
Urban	95.1	686	59.7	498	80.2	1184
Rural	94.7	1034	60.8	2292	71.3	3326
**Wealth:**	*		†		‡	
Poorest	95.5	149	54.0	832	60.3	982
Second	93.2	205	60.0	643	68.0	849
Middle	90.6	292	62.5	566	72.1	859
Fourth	96.6	439	66.6	494	80.7	932
Richest	96.1	634	67.6	254	87.9	889
**Antenatal visits:**			‡		‡	
None	92.1	107	49.2	870	53.9	977
1	95.1	213	62.8	602	71.2	815
2	94.8	300	64.8	428	77.2	728
3	96.6	285	72.0	313	83.7	598
4+	94.6	815	66.1	576	82.8	1392
**Total**	94.9	1719	60.6	2789	73.7	4510

*P < 0.05.

†*P* < 0.01.

‡*P* < 0.001.

## DISCUSSION

Monitoring ENC is needed to identify programmatic coverage gaps and to provide an empirical basis for policy, accountability and investment to improve newborn survival and care. The renewed emphasis on newborn survival on the global agenda has led to the inclusion of new questions and modules on ENC in survey programs for the collection of data on key indicators in a systematic manner. For instance, in the latest round of MICS surveys, launched in late 2016, questions on ENC including thermal practices, cord care and skin-to-skin contact are included for countries to use. Changes have also occurred for the DHS surveys, which has an optional newborn care module with similar topics.

Levels of drying are remarkable similar in the two countries with nearly all newborns being dried. However, bathing practices differ in the two countries, with newborns in Malawi being bathed much earlier than those of Bangladesh. These levels seen in Malawi are much lower than those seen in Uganda in other literature [12]. The analysis also shows that skilled providers are important for the delivery of timely drying in both countries and drying in Malawi. However, given that a sizeable proportion of births occur outside of facility settings, it would be useful to ensure that unskilled providers are able to provider these two elements of thermal care. This recommendation is supported by the 2009 WHO/UNICEF joint statement on home visits for newborns, wherein both skilled and unskilled providers are endorsed for certain elements of care, including thermal care, following the time of birth [17].

Our results show that the measurement approaches used in these two surveys are not standardized. This is to be expected as these are initial efforts to measure these variables in the survey programmes. Drying data for the two surveys are largely incomparable. While the Malawi survey data allow a category of ‘immediate drying’, this category also includes cases of drying within 1 hour. The Bangladesh survey provides response categories using ranges of minutes and no ‘immediate’ drying category. As such, both cases do not allow an easy calculation of the ‘immediate drying’ indicator, which in part reflects the lack of clarity of what constitutes ‘immediate’. These concerns are largely addressed in the latest round of MICS and the DHS optional module on newborn care. In MICS6, the relevant question asks if the newborn was wiped or dried soon after birth, while the DHS asks if the newborn was wiped dry within a few minutes. It is of course necessary to note that both question approaches allow a certain level of subjectivity to measure the immediacy of drying, an approach that is necessary until there is an agreed definition of what ‘immediate drying’ refers to.

With reference to newborn bathing, the major methodological issue is that the cut-off for the indicator is for values greater than 24 hours. Both surveys show heaping of data at the 24-hour mark, which is used as a cut-off for indicator calculation. Similar heaping of data exists across different health and nutrition indicators such as low birth weight (at 2500 g). Due to this issue, in the latest round of MICS, after the question on bathing is asked, an addition probe is activated if the respondent says that the newborn was bathed on the 1 day/24-hour mark to ascertain if the bath took place before, after or on that exact moment. A similar approach can be useful for surveys using the DHS approach. We also detect that in Malawi an important proportion of cases was not bathed, but in Bangladesh, there were much fewer cases. It would be important to monitor this proportion in future surveys and to understand the circumstances that produce this outcome.

There are important limitations in this paper that should be considered when interpreting the results. Data on ENC practices used in the analysis are based on mother’s recall of care provided to the newborn soon after birth. Apart from recall, women not know if certain events have occurred or the exact timing, especially in the case of C-sections, where the mother and newborn may be quickly separated. This may explain why women who had a C-section reported lower levels of drying. As with other measures based on mother’s recall, this could have led to differential recall bias and may not entirely reflect the level of quality of care in facilities [18]. This is particularly the case for interventions that occur during the postpartum period as it is an intense moment for mothers. The Malawi MICS 2014, newborns who were not dried were skipped out of the question on bathing which can potentially under-estimate the indicator level, given that some newborns who are not dried could have a bath later than the 24-hour period. Due to the questions asked, a comparable measure of immediate drying could not be calculated. As such, the analysis in this paper focusses on newborns who were ever dried or not.

Our findings shed light into gaps on two essential yet simple newborn care interventions, which at low cost, can help maintain the newborn in a stable condition. The results also indicate potential areas of intervention for training up of staff for implementing drying and bathing in these countries. Further research is required to study the results of the new MICS and DHS approaches to measuring drying and bathing in different countries to ensure that the intention of these questions are properly reflected.
